# Expanded Radiosurgery Capabilities Utilizing Gamma Knife Icon™

**DOI:** 10.7759/cureus.13998

**Published:** 2021-03-19

**Authors:** Jameson T Mendel, Samuel Schroeder, Aaron Plitt, Ankur Patel, Mindy Joo, Strahinja Stojadinovic, Tu Dan, Robert Timmerman, Toral R Patel, Zabi Wardak

**Affiliations:** 1 Radiation Oncology, Rio Grande Urology, Dallas, USA; 2 Radiation Oncology, UnityPoint Health, Des Moines, USA; 3 Neurological Surgery, University of Texas Southwestern Medical Center, Dallas, USA; 4 Neurosurgery, Baylor Scott & White Health, Dallas, USA; 5 Radiation Oncology, University of Texas Southwestern Medical Center, Dallas, USA; 6 Neurosurgery, University of Texas Southwestern Medical Center, Dallas, USA

**Keywords:** gamma knife icon, stereotactic radiosurgery, stereotactic radiotherapy

## Abstract

The indications and techniques for the treatment of intracranial lesions continue to evolve with the advent of novel technologies. The Gamma Knife Icon™ (GK Icon™) is the most recent model available from Elekta, providing a frameless solution for stereotactic radiosurgery. At our institution, 382 patients with 3,213 separate intracranial lesions have been treated with frameless stereotactic radiotherapy using the GK Icon. The wide range of diagnoses include brain metastases, meningiomas, arteriovenous malformations, acoustic neuromas, pituitary adenomas, and several other histologies. The ability to perform both frame and frameless treatments on the GK Icon has significantly increased our daily volume by almost 50% on a single machine. Although the frameless approach allows one to take advantage of the precision in radiosurgery, the intricacies regarding treatment with this frameless system are not well established. Our initial experience will help to serve as a guide to those wishing to implement this novel technology in their practice.

## Introduction

In the 1950s, Lars Leksell established the basic principles surrounding stereotactic radiosurgery (SRS), eventually leading to the development of the Gamma Knife (GK) system [1]. Treatment planning software has evolved with the ever-accelerating computational developments over the past decades. However, the underlying principles of SRS remain relatively unchanged. A key component of the delivery of radiosurgery is the management of intrafraction tumor motion, which has been traditionally accomplished through rigid immobilization. As with previous models (U, B, C, and 4C), the automated GK Perfexion™ uses a stereotactic frame for rigid immobilization and localization. The GK Icon™ (Elekta, Stockholm, Sweden) is the sixth-generation and most recent commercial model. This latest upgrade includes a cone-beam computed tomography (CBCT) module, permitting non-invasive immobilization utilizing frameless treatments without compromising sub-millimeter accuracy [2,3]. Frameless stereotactic radiotherapy (SRT) allows for custom-tailored utilization and opens many new indications for treatment on the GK system. Given the novelty, the intricacies, regarding logistics, patient selection, and appropriate treatment regimens with frameless systems, are not well established. In this article, we describe one of the first experiences with frameless treatments on the GK Icon.

## Materials and methods

The GK Icon continues to be one of the most robust and reliable stereotactic delivery systems. Similar to the Perfexion model, 192 cobalt-60 sources are arranged in a conical shell, distributed within eight sectors, allowing for a nearly 2π geometry. Each sector has 24 sources, which can be blocked or positioned above 4-, 8-, or 16-mm diameter collimators, resulting in converging gamma beams of corresponding diameters at the isocenter. This source arrangement produces an ellipsoidal dose distribution with rapid three-dimensional (3D) falloff, which is the sharpest in the craniocaudal direction. We recommend methodical evaluation of all imaging planes with careful attention to the craniocaudal dimension using coronal and sagittal views, given the rapid dose fall-off in this orientation.

**Figure 1:**
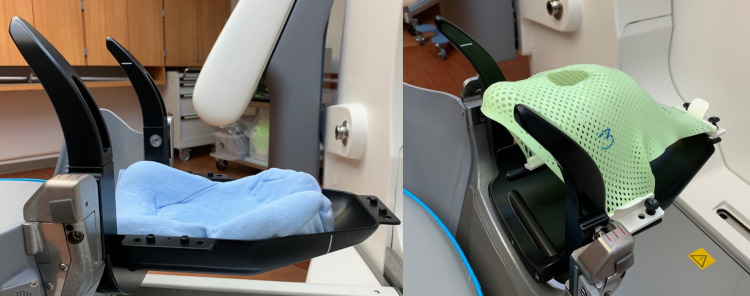
Mask adapter for GK Icon. The water-activated polystyrene cushion (left) is custom shaped to a suitable head positioning for the patient. The cushion rests within the mask adapter, which has an indexing indentation visible in the right image to hinder cushion translation along the craniocaudal (z) axis. White lines on the adapter define the optimal treatment space for head positioning. A three-point aquaplast mask (right) is used for head immobilization. A submental mask lip helps prevent uncomfortable mask fitting. A nose opening is formed, allowing placement of an optical marker. GK = Gamma Knife

During frameless simulation, the patient’s head is first positioned on a dampened moldable polystyrene cushion. Head positioning during this step is critical to prevent collisions in tumors that are polarized in the anterior, posterior, superior, or inferior locations in the cranial vault. The GK machine is designed such that the patient moves around a fixed isocenter, driven by the movement of the couch. Proper head positioning to minimize shifts from this isocenter is important to prevent collisions and is described in further detail during discussion of frameless treatment. A polystyrene cushion (Figure 1, left) is custom-shaped to allow for a suitable patient head positioning. Once set, an Icon-specific, oven-heated, three-point thermoplastic mask is created for immobilization (Figure 1, right). A reference CBCT scan is then acquired to define the stereotactic treatment space. Planning images (magnetic resonance imaging (MRI)/computed tomography (CT)) are registered to the reference CBCT. Daily and intrafractional acquisition of a pre-treatment lower-contrast CBCT allows for adjustment of minor daily positional changes in the X, Y, and Z directions. Note that standard diagnostic imaging is often not appropriate for GK treatment planning. Planning images require volumetric imaging (1-mm slice thickness, i.e., spoiled gradient-recalled echo [SPGR], magnetization-prepared rapid gradient echo [MP-RAGE]), or equivalent with scanner-specific geometric validations and quality assurance testing in accord with published guidelines for SRS. At our institution, we use a 3D, T1-weighted, post-contrast, magnetization-prepared, 180-degree radiofrequency pulses and MP-RAGE with fat saturation (FS) sequence for planning. Especially for histologies exhibiting rapid growth, planning images should be acquired close to the treatment date to ensure negligible tumor growth and/or surrounding parenchymal changes. In a recent study evaluating the impact of treatment delay in patients with brain metastasis, 78% of stereotactic targets that were delayed for more than seven days from imaging required target adjustments, the majority from tumor growth [4]. Use of follow-up diagnostic imaging for treatment may ultimately improve workflow efficiency, provided diagnostic imaging is acquired with volumetric imaging. However, pre-authorization for treatment often delays its start beyond the optimal time-frame.

The GK Icon uses a high-definition motion management system for intra-treatment motion monitoring. The system consists of a pair of infrared stereoscopic cameras on a movable arm attached to the couch, a set of four reference markers integrated within the mask adapter, and an adhesive optical marker placed on the patient’s nose. Intrafraction thresholds of 1 to 3 mm (default 1.5 mm) are specified depending on lesion location or physician preference. If the threshold is breached, treatment is interrupted. The treatment can be resumed if the patient returns within the set limit or by obtaining a new reference position with additional CBCT to verify patient setup. When treating near critical structures, a planning risk volume may be utilized to maximally limit high-dose overlap, as well as the application of stringent threshold settings. It is important to note that motion using this system is used as a surrogate for overall motion stability rather than direct target motion. Changes in nasal breathing patterns and movement of the nose may have negligible effects on tumor motion, and each situation should be evaluated on a case-by-case basis to set the appropriate threshold. Despite the use of varying motion threshold settings and no planning target volume (PTV), we have yet to detect a targeting error with frameless treatments.

At University of Texas Southwestern (UTSW), frameless GK SRS is commonly used. From January 2017 to February 2020, 382 patients with 3,213 separate intracranial lesions were treated with frameless SRT using the GK Icon. Diagnoses included 312 brain metastases, 29 meningiomas, eight arteriovenous malformations, 15 acoustic neuromas, two pituitary adenoma, and 16 miscellaneous histologies. In the article, we describe the treatment logistics and common diagnoses managed utilizing the frameless technique on the GK Icon.

## Results

Brain metastases

Brain metastases develop in 3-50% of patients with a cancer diagnosis [2,5]. We prefer SRS over whole brain radiotherapy in patients with limited brain metastases as it is associated with less cognitive toxicity and improved quality-of-life [6,7]. Prescription doses vary based on the size and number of lesions. Patients are prioritized for enrollment in our in-house dose escalation protocol. Off-protocol, the most commonly used maximum single-fraction doses are extrapolated from the Radiation Therapy Oncology Group (RTOG) trial 90-05: lesions ≤2 cm receive 24 Gy, 2.1-3 cm receive 18 Gy, and 3.1-4 cm receive 15 Gy [8]. Most practitioners would recommend against treating lesions >4 cm with SRS due to concerns of increased toxicity. Traditionally, only patients with one to three brain metastases were considered candidates for radiosurgery. However, as data continue to emerge in patients with up to 10 brain metastasis or more, this convention has been challenged [9]. In a recent multi-institutional prospective observational study of SRS for multiple brain metastases, Yamamoto et al. demonstrated non-inferior survival in patients with five to ten brain metastasis compared to patients with two to four brain metastasis [10]. Data were further expanded to a case-matched study comparing patients with <10 versus ≥10 metastases. Interestingly, neurological death-free survival, local recurrence, repeat SRS for new lesions, and SRS-related toxicity were not inferior for patients with ≥10 brain metastases [11]. The GK Icon will help facilitate stereotactic treatment of increasingly more brain metastases with evolving treatment paradigms. Currently we are enrolling patients on an in-house protocol for those with less than six lesions with no upper maximum number of lesions to assess disease and neurocognitive outcomes.

Stereotactic Radiosurgery, Stereotactic Radiotherapy, and Distributed Treatments

Frameless treatments on the GK Icon now allow for SRS, SRT, and/or distributed treatments. SRS traditionally refers to a single cranial radiotherapy treatment where all lesions receive the full Rx dose in one session. SRT refers to the division of total Rx dose over several treatment sessions where all lesions receive fractionated Rx dose per treatment session. “Distribution” denotes treatment of multiple lesions over the course of several treatment sessions where selected lesions receive the full Rx dose within a session. Due to software limitations, frameless treatments require additional planning and coordination which are not obvious to the first-time user.

Frame Versus Frameless Treatments

When selecting patients for frameless treatment, one must consider the location, number, and size of lesions. We recommend using frame-based treatments for single sessions lasting more than one hour or lesion(s) <2-3 mm from a critical structure. Patients receiving SRS for functional disorders, such as trigeminal neuralgia, are also recommended to have rigid immobilization due to the doses used. Distributed or fractionated approaches can improve convenience as well as mitigate normal tissue toxicity in patients with numerous (>eight) or large (>2 cm) lesions. Fortunately, in cases requiring distribution or fractionation after frame placement, transition to frameless treatment is generally straightforward as the initial planning MRI can be registered to new CBCT coordinates.

Estimating treatment time and tolerability is more an art than science. While patients with a few small-volume brain metastases in non-eloquent locations may seem natural candidates for frameless treatment, their perceived tolerability has been a reliable indicator of success. We employ a patient-centered approach regarding selection of mask versus frame-based treatment. The ideal mask patient is relaxed, alert, and able to lie still for at least 30-60 minutes. Those who are restless, agitated, or fatigued are not optimal candidates as the ability to remain motionless requires patient participation. For this reason, sedation/anxiolytics are not recommended during treatment. Although frameless treatments have similar accuracy as frame-based treatments, overly stringent motion thresholds may lead to increased total treatment time due to repeated patient repositioning [2,3]. Additionally, source activity, shot efficiency, and total dose delivered should be taken into consideration when estimating overall treatment time.

Simulation and Treatment Planning

**Figure 2:**
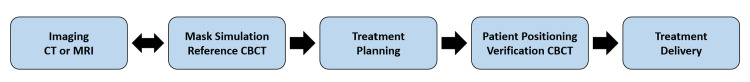
Workflow variations for frameless treatment delivery with Icon. CT = computed tomography; MRI = magnetic resonance imaging; CBCT = cone-beam computed tomography

The GK Perfexion solved many positioning challenges by incorporating the adjustable gamma angle (70°, 90°, and 110° options). Frameless treatments require the use of a rigid head support that is locked at a 90° gamma angle. Figure 2 shows the workflow for frameless treatment with Icon. During mask simulation, it is valuable to communicate distribution of targets within the brain to facilitate optimal head positioning. A neutral head position is typically recommended. However, possible clearance issues may be mitigated by building up superiorly or flattening the head cushion. Anterior lesions are best positioned with a neutral head positioning. Low-lying posterior fossa lesions typically require chin-down positioning. Additionally, low-lying posterior fossa lesions benefit from patient shoulder positioning against support pillars for adequate coverage. Lesions distributed throughout the anterior frontal lobes and posterior fossa may require simulation in both neutral and chin-down positions. Brain metastases are best visualized with a 1-mm slice T1-post MP-RAGE with 3D reconstructions. The addition of a 1-mm 3D fast (turbo) spin-echo sequence (sampling perfection with application-optimized contrast using different flip-angle evolution [SPACE]/CUBE/volume isotropic turbo spin echo acquisition [VISTA]) can be useful for patients with numerous punctate brain metastases. Table 1 lists the MRI parameters for T1 MP-RAGE FS post-contrast and SPACE sequences on our 3T magnet. The 3D fast spin-echo sequence aids visualization, but it is not highly specific for brain metastases. Therefore, correlation with the planning post-contrast scan is always recommended. To ensure proper dose calculations, the planning MRI should extend at least 4-5 cm inferior from the lowest lesion or to the bottom of C2. Insufficient scan length may produce inadequate skin mapping, rendering inaccurate dose calculations. If the planning MRI is inappropriately acquired, fusion of a previous diagnostic MRI may aid in cranial or caudal skin mapping.

**Table 1:** Planning imaging MRI parameters. ms = milliseconds; mm = millimeters; TR = repetition time; TE = time of echo; MP-RAGE FS = magnetization-prepared rapid gradient echo with fat saturation; SPACE = sampling perfection with application-optimized contrasts using different flip angle evolution; MRI = magnetic resonance imaging

Sequence	TI (ms)	TR (ms)	TE (ms)	Matrix	Voxel size (mm^3^)
T1 MP-RAGE FS POST	1,140	2,260	3.4	256 × 256	1 × 1 × 1
SPACE		650	20	256 × 256	1 × 1 × 1

Fractionation

With improvement in systemic options, maintaining intracranial control while mitigating neurocognitive toxicity is now a major goal in patients with intracranial metastases [12,13]. While surgical resection remains an excellent option in patients with large brain metastases, not all patients are ideal surgical candidates [14]. SRS and SRT offer an alternative treatment strategy to whole brain with superior durability and cognitive outcomes [7,9,10]. For brain metastases that are 3-4 cm in largest dimension, 15 Gy represents the maximum safely deliverable prescription dose with SRS [8]. To mitigate toxicity and increase total dose, fractionation with GK has been explored. Higuchi et al. reported local control of 76% at one year in 43 patients receiving three fractions of 10 Gy every two weeks with complication rates of <5% [15]. Since then, several groups have demonstrated comparable results over similar treatment schedules [16,17]. Shortly after introduction of the GK Perfexion, Elekta offered an extended bite-block palatal vacuum immobilization system (i.e., eXtend). However, it has not found widespread use due to technical complexity. Fortunately, frameless treatment on the GK Icon eliminates many technical issues, allowing for SRT of large brain metastases.

**Figure 3:**
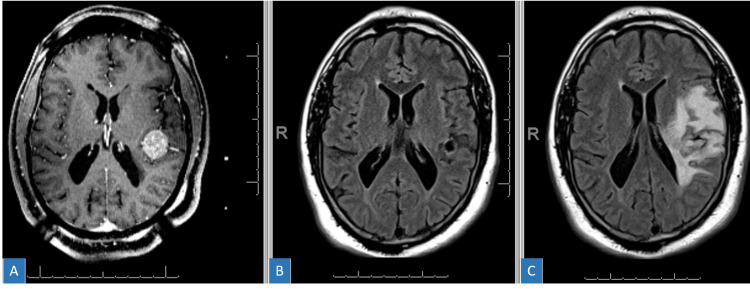
Radiation necrosis after 27 Gy in three fractions. (A) Pre-treatment planning T1 with contrast MRI. (B) Three-month post-treatment T2 FLAIR MRI with excellent response and minimal surrounding edema. (C) Six-month post-treatment T2 FLAIR MRI with clinically symptomatic edema. FLAIR = fluid attenuated inversion recovery; MRI = magnetic resonance imaging

**Figure 4:**
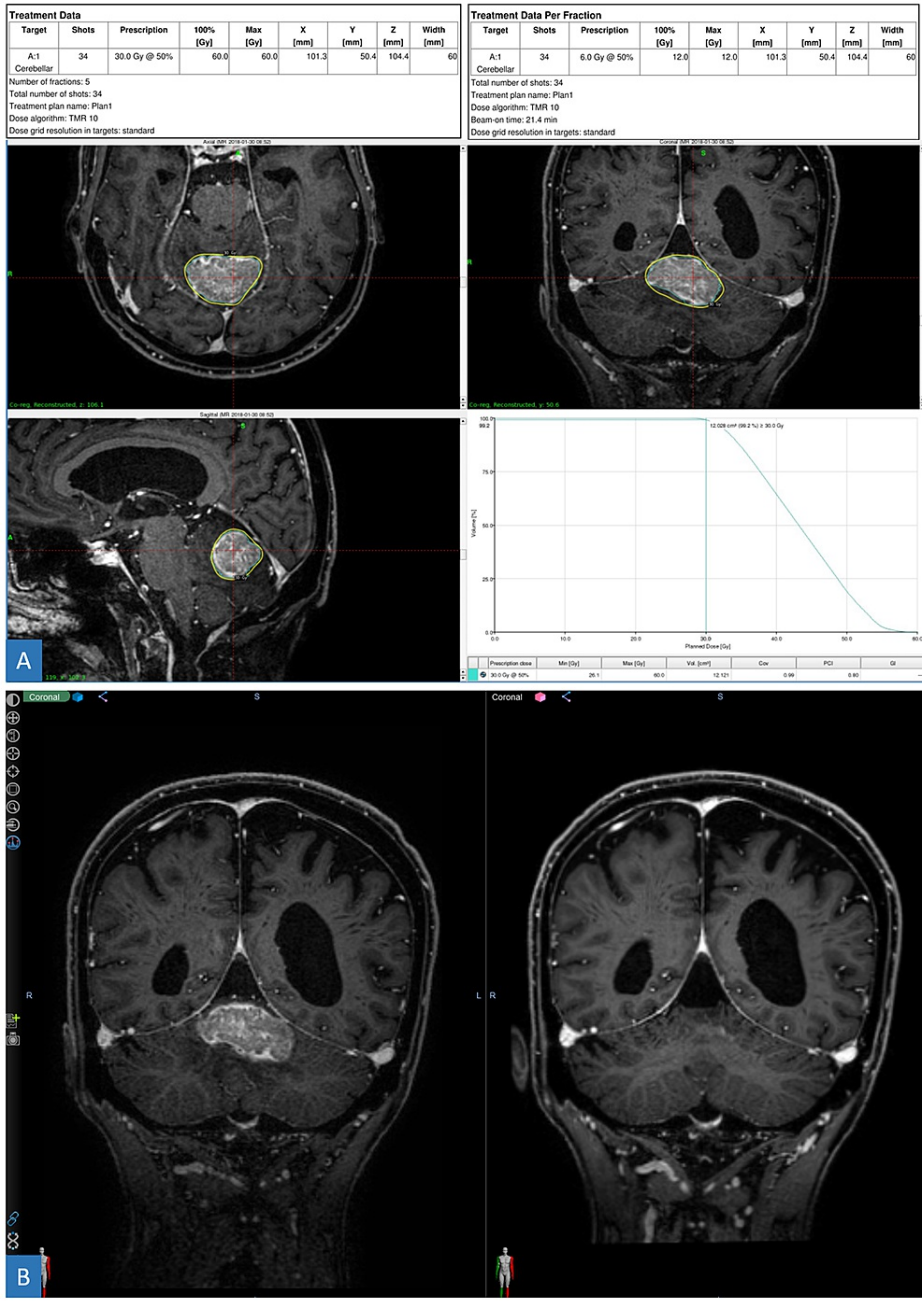
72-year-old male with brain metastasis of prostate adenocarcinoma primary. (A) Axial (upper left), sagittal (lower left), and coronal (upper right) views of a large vermian lesion receiving 30 Gy in five fractions via GK radiosurgery. (B) T1 post-contrast coronal views of pre-treatment (left) and three-month follow-up (right) scans demonstrating complete resolution of enhancement. GK = Gamma Knife

**Figure 5:**
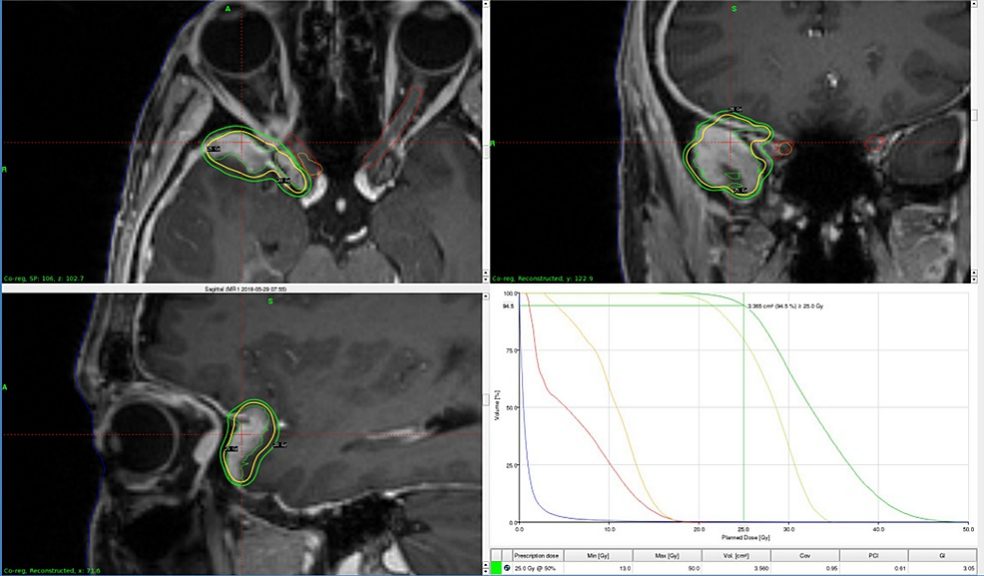
Dural-based metastasis in the right anterior temporal region that was treated with 25 Gy in five fractions on the GK Icon. GK = Gamma Knife

In an early UTSW institutional analysis of 29 patients with 43 separate intracranial lesions, receiving mask-based SRT with either 20-30 Gy in five fractions or 27 Gy in three fractions with no margin, local control was >90% at one year [15-17]. Radiation necrosis occurred in all patients who received 27 Gy in three fractions, with one requiring therapeutic resection (Figure 3). Out of concern for increased toxicity, we have abandoned this three-fraction regimen and recommend exercising caution with translating LINAC-based dosing schemes to the GK Icon. Incidence of local failure for all treated lesions was 9% at one year, comparable to previous series. Currently at our institution, most brain metastases >2.5 cm receive SRT to 30 Gy (range 20-30 Gy) in five fractions over the course of two weeks (Figures 4, 5).

Distribution

**Figure 6:**
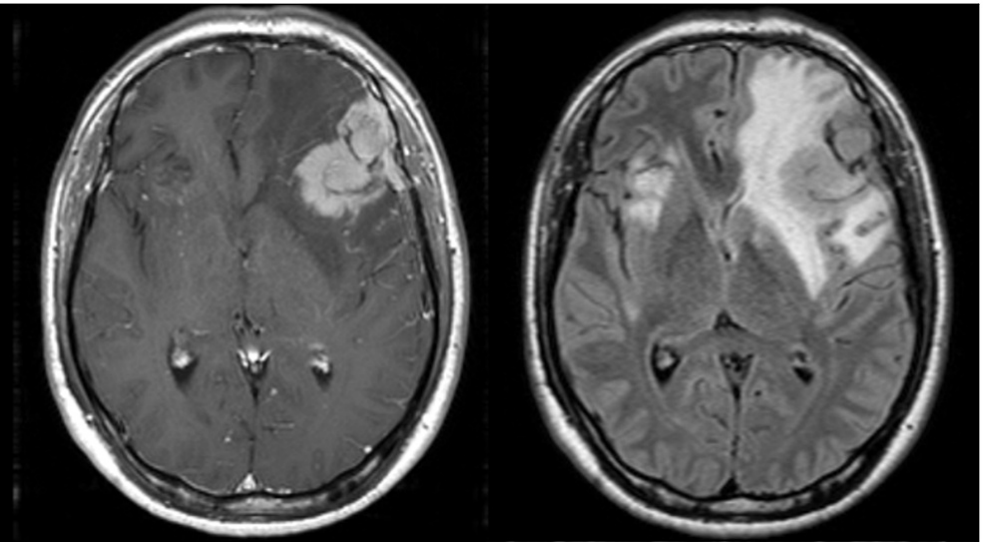
Concerns with multiple brain metastases. T1 post-contrast MRI with two left frontal brain metastases in close proximity (left) with significant associated edema after treatment on T2 FLAIR MR (right). MRI = magnetic resonance imaging; FLAIR = fluid attenuated inversion recovery

**Figure 7:**
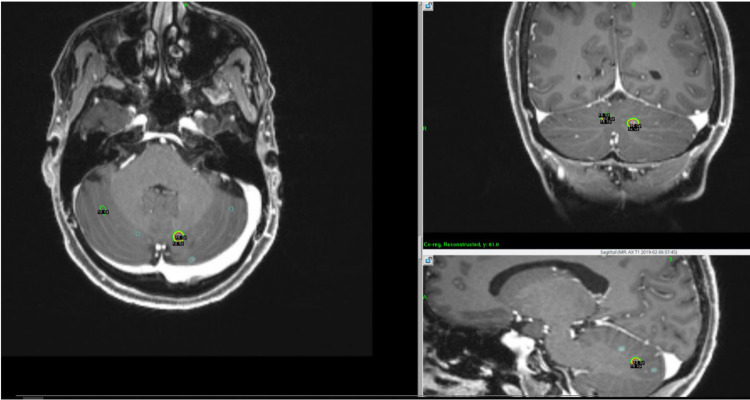
Distributed treatment. Dose interaction between shots can contribute to treatment-related toxicity. Distributed treatment on separate days may help to facilitate repair within intervening normal brain parenchyma. The blue contours represent lesions that were previously treated in a different session.

SRS to numerous brain metastases in a single frame-based session poses several challenges, including treatment duration and proximal lesion dose overlap. Due to these issues, most radiosurgeons consider 20-30 lesions beyond the upper limit of treatable brain metastases in a single frame-based session [18,19]. Frameless treatments overcome this challenge by distributing treatments over the course of days to weeks. At UTSW, from January 2017 to November 2018, 1,139 brain metastases in 56 patients (range three to 81 lesions per course) received distributed frameless SRS for a total of 66 courses in two to five sessions per course. In general, treatment times were limited to <60 minutes for each frameless session (range 14-138 minutes). In some cases, a low-dose overlap was noted in proximal lesions. If treated in the same session, severe edema and necrosis could occur around the proximal lesions, as shown in Figure 6. Out of concern for increased toxicity, lesions in close proximity are initially identified and planned for treatment in separate sessions (Figure 7). The current software does not offer a straightforward system for organization of distributed treatments. We delineate and list potentially troublesome lesions and then distribute clusters on separate treatment days. Once mapped, additional lesions from six different anatomic sectors (left/right anterior/posterior hemisphere and left/right cerebellum) are chosen to further minimize low-dose overlap within each sector during each treatment session (Table 2).

**Table 2:** Designing distributed treatments. Concerning lesions (lesion pairs two and seven, 10 and 13, and 17 and 19) are initially identified and distributed. Additional lesions are then evenly distributed throughout the treatment course. Planning may be accomplished either by generating a “master plan” or separately planning each consecutive treatment. If a master plan is used, individual shot coordinates must be manually typed for all but the first treatment.

Treatment day	1	2	3	4	5
Lesions	1	3	5	7	9
	2	10	6	8	13
	4	12	19	11	14
	17	18	20	15	16
	21	22	24	22	23
	25	27	26		

In our early unpublished experience, the majority of lesions treated in a distributed fashion were small (<2 cm), and received 13-18 Gy that was prescribed to the 40-70% isodose line with a local control of about 75% at the last follow-up. Although well below the RTOG 90-05 dosing schemes, prescription doses of 13-15 Gy allow for reduced treatment times and may help to avoid treatment-related toxicities. Moraes et al. reported similar findings after analyzing 1,533 brain metastasis treated with GK 4C and Perfexion. In this study, lesions ≤1 cm receiving 12-15 Gy seemed to have no discernable difference in local failure compared to those receiving 21-25 Gy [20]. For patients whose disease recurs after low-dose SRS, salvage radiosurgery is safe and often successful.

Troubleshooting Fractionation With Distribution

**Figure 8:**
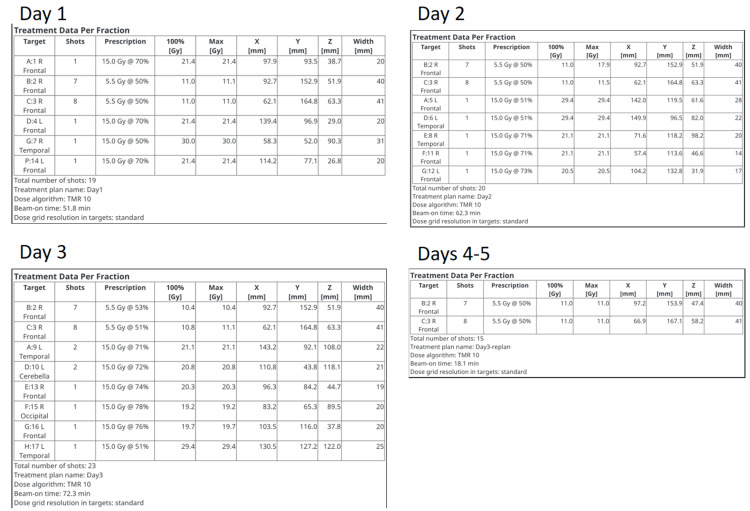
Treatment logistics for distributed and fractionated lesions.

Patients with large brain metastases often present with synchronous punctate metastases. In this situation, we favor 20-30 Gy in five fractions for lesions >2.5 cm and 13-15 Gy to additional smaller lesions distributed over the course of the planned fractionation schedule. Figure 8 presents an example of a patient with 17 brain metastases, two of which received 27.5 Gy in five fractions while the remaining lesions were distributed which received 15 Gy each. Early on in our practice, a new plan was generated for each treatment day by utilizing the “replan” function in the treatment planning system (TPS). The “replan” inherently copies the fractionated lesion(s) forward and allows deletion of previously treated single-fraction lesions and addition of new ones. However, this way the TPS cannot account for dose contributions from the subsequent fractions/distributions and some of the originally treated lesions may receive more total dose than intended. Therefore, we have transitioned to the creation of an initial “master plan” for accurate dose assessment. In this scenario, the “replan” function is still utilized. However, due to current software limitations, shots coordinates must be manually entered for all but the first treatment, and Rx isodose lines have to be manually adjusted to match the treatment times in the master plan. This requires meticulous attention to detail to avoid human errors.

Additional metastases are often identified on the high-resolution planning MR when pursuing frame-based treatment. Should the number of metastases exceed the single-treatment time tolerance of the patient/practitioner, transition to frameless treatment offers an easy solution. Patients simply undergo simulation for frameless treatment with registration of the planning MRI to the simulation CBCT. This workflow may be useful for patients with multiple metastases and few in critical locations (e.g., the brainstem). For lesions situated within the brainstem, we prefer to use frame-based treatment to a dose of 13 Gy, prescribed at the 50% isodose line, although greater doses may be considered [21].

Meningioma

**Figure 9:**
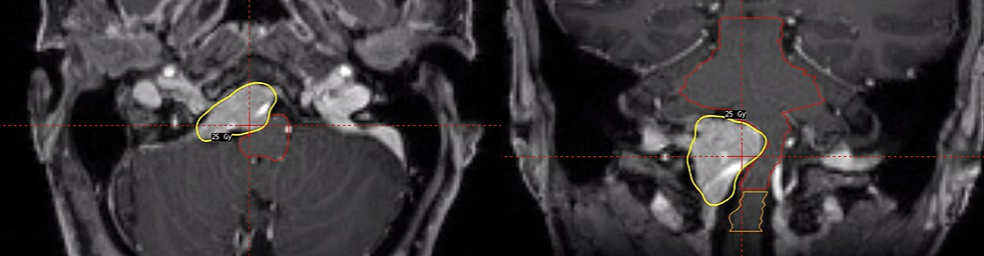
Axial (left) and coronal (right) T1 post-contrast planning MRI of a skull-based WHO grade I meningioma that was treated with 25 Gy in five fractions on the GK Icon. MRI = magnetic resonance imaging; WHO = World Health Organization; GK = Gamma Knife

**Figure 10:**
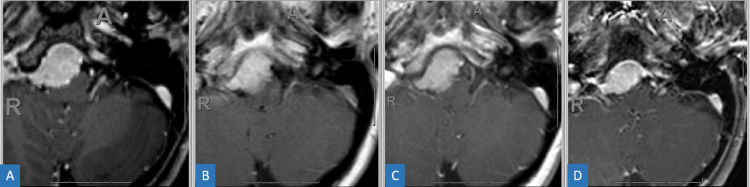
Pre-treatment (A), four-month (B), one-year (C), and two-year (D) post-treatment axial T1 post-contrast MRI of a skull-based WHO grade I meningioma. Notice the hazy tumor edges on four-month and one-year follow-up scans with eventual contraction of the tumor at two years. MRI = magnetic resonance imaging; WHO = World Health Organization

Meningioma is the most common primary brain tumor in adults. For accessible lesions, maximal safe surgical resection is considered the standard of care. Although patients are commonly treated based on the risk groups defined in RTOG 0539 [22], we prefer to treat progressive World Health Organization (WHO) grade I meningiomas with 15-16 Gy and higher-grade meningiomas in a single fraction or multiple fractions, prescribing 30 Gy and five fractions. Although toxicity predictions and normal tissue constraints continue to evolve, tumor location and tumor surface area may prohibit safe SRS [23]. In these instances, we prefer SRT with 25 Gy in five fractions in an attempt to mitigate toxicity. For example, Figure 9 shows a skull-based WHO grade I meningioma abutting and distorting the brainstem. As treatment with SRS exceeded brainstem constraints, the patient received 25 Gy in five fractions on the GK Icon. The common evolution of treatment response is shown in Figure 10. Thus far, no toxicity or tumor recurrence has been noted using this technique at our institution.

Vestibular schwannoma

**Figure 11:**
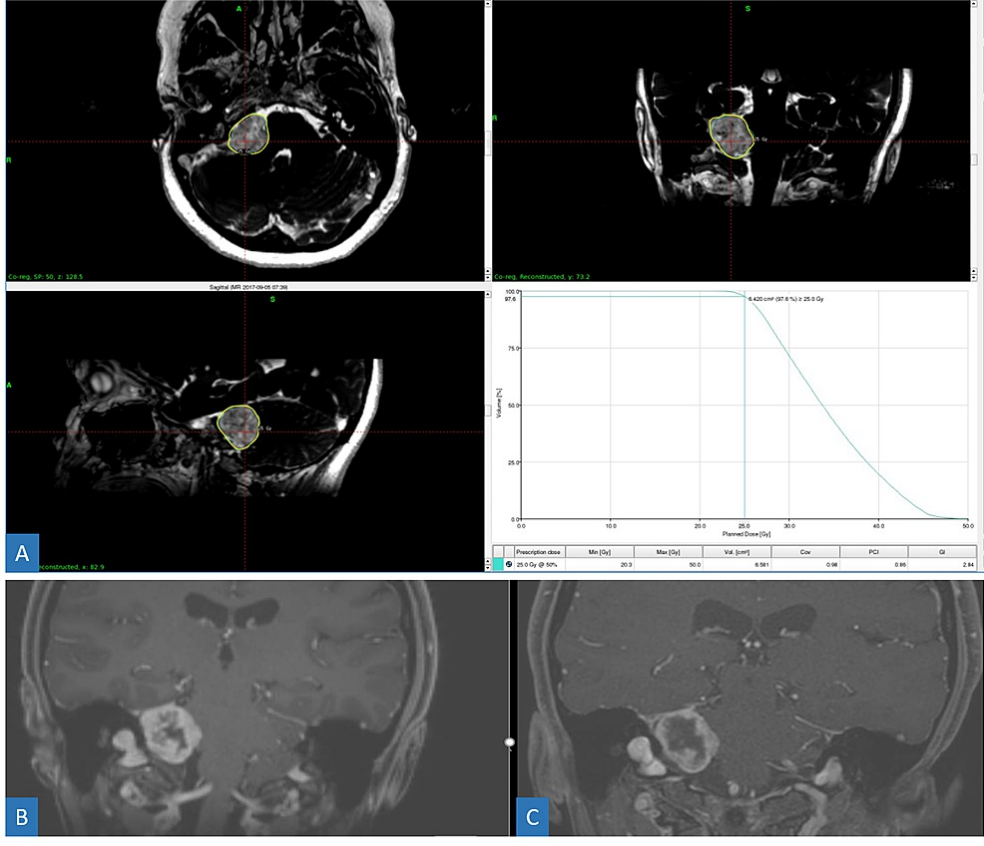
Patient with vestibular schwannoma. Treatment plan (A) for a right-sided vestibular schwannoma which received frameless treatments of 25 Gy in five fractions on the GK Icon. Pre-treatment (B) and post-treatment (C) coronal T1 post-contrast MRI of a right-sided acoustic neuroma. Notice the dramatic increase in central necrosis post-treatment, characteristic of an excellent treatment response. GK = Gamma Knife; MRI = magnetic resonance imaging

Vestibular schwannomas or acoustic neuromas are benign tumors that most commonly arise from the Obersteiner-Redlich zone, where glial cells transition to Schwann cells, in the vestibulocochlear nerve. At our institution, we tend to favor surgical removal in young patients or those with large lesions. Over the years, SRS has established itself as a less invasive and equally efficacious treatment option [24]. Dose and fraction schemes for stereotactic treatment vary depending on the size and location of the tumor but are typically below the threshold for significant neural and brainstem toxicity. Typical stereotactic doses range between 12 and 13 Gy [25]. Of those treated with frameless SRS to 13 Gy at our institution, we have not anecdotally noted increased recurrence or toxicity. Figure 11 shows a patient example of frameless treatment with Icon.

Arteriovenous malformation

**Figure 12:**
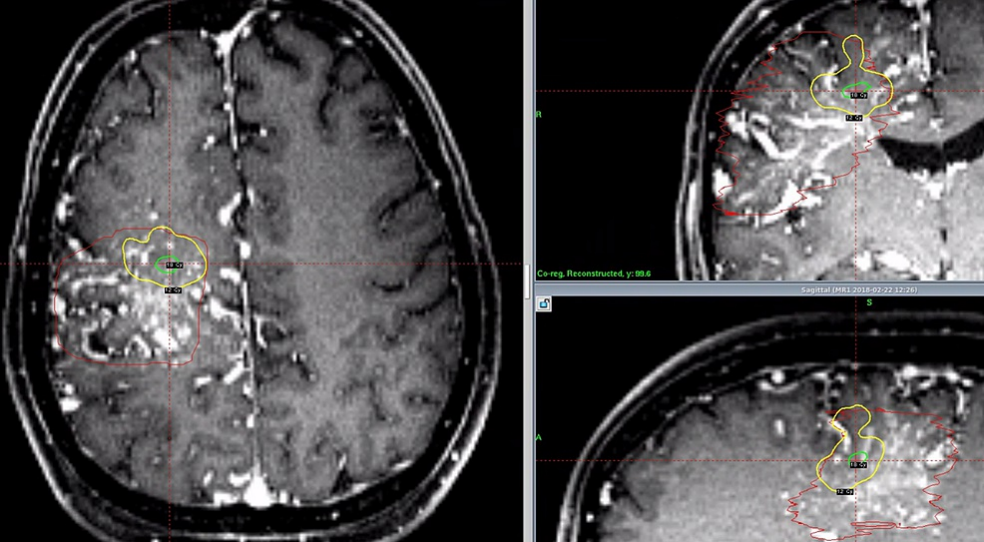
Axial (left), coronal (upper right), and sagittal (lower right) T1 post-contrast MRI of a large right-sided frontoparietal AVM treated with staged SRS. This AVM was divided into eight slices, and each received 12 Gy on separate days. MRI = magnetic resonance imaging; AVM = arteriovenous malformation; SRS = stereotactic radiosurgery

Arteriovenous malformations (AVMs) are sporadic congenital vascular abnormalities consisting of arteries with direct connections to the draining vein. While most lesions are clinically silent, up to half of symptomatic patients present with acute intracranial hemorrhage. AVMs with prior hemorrhage, deep venous drainage, diffuse morphology, and deep location are at an increased risk of hemorrhage. Depending on the location and angioarchitecture of AVM, treatment options include the following, either alone or in combinations of: medical management, surgery, radiosurgery, embolization, and/or observation [26]. For poor surgical candidates or those with incomplete embolization, SRS offers a non-invasive and effective treatment option. To ameliorate this, most institutions now use a combination of two-dimensional angiography and computed tomography angiography and/or magnetic resonance angiography [27]. Based on the literature, prescription dose ranges from 18 to 24 Gy for AVM radiotherapy. At UTSW, we prefer to use 18-20 Gy given the plateau in response at this dose level. Large AVMs typically receive a staged stereotactic approach on the GK [28-30]. Frameless treatments on the GK Icon permit staged stereotactic treatments without multiple frame placements. However, planning remains complex given current limitations of the GK TPS software. Initially, a master plan with an Rx dose of 20 Gy is created for the entire target. Next, the AVM is typically divided into two to eight sub-targets or slices (5-6 cc each). The shots within each sub-target are identified, and a new lower dose of 12 Gy dose is prescribed. The shots’ coordinates must be manually typed for all but the first treatment, and the Rx isodose lines have to be manually adjusted to match the treatment times in the master plan. This is an arduous process, requiring meticulous record keeping and multiple checks. Each sub-target receives a single treatment on separate days over the course of weeks to months, depending on the size of the lesion and physician preference. Figure 12 is an example of a patient with a large AVM treated with staged treatment. To reduce treatment toxicity, only one slice is treated per treatment session. Obliteration of the nidus can take three to five years, after which the risk of hemorrhage is reduced by up to 88% although never completely eliminated.

## Discussion

The ability to perform both frame-based and frameless treatments on the GK Icon has substantially impacted our clinical workflow and practice. The daily volume of frame-based GK treatment is inherently capacity-constrained because of factors including limited Leksell Stereotactic Frames and nursing as well as MRI availability. Frameless treatment workflow alleviates all of these critical constraints and thus allows for a greater number of patients to receive treatment each day, similar to a conventional linear accelerator radiation treatment machine. Based on our ability to utilize frameless planning for patients where other stereotactic treatment platforms (i.e., CyberKnife radiosurgery) were previously used, we increased the total number of patients treated during the first full fiscal year after installation by 47% (403 patients in total). To further enhance clinical efficiency for patients with brain metastases, the GK-specific T1 post-contrast MRI protocol can be added to follow-up scans and be used for immediate planning/treatment if new metastases are detected. Such an approach provides the favorable dosimetry of GK treatments in an expedient manner.

## Conclusions

Implementation of the GK Icon has allowed for both fractionated and distributed treatments that were not possible on previous GK iterations. Our initial experience with treating a wide range of intracranial pathologies using frameless approaches has been favorable and takes advantage of the excellent conformality offered by GK. Although our initial experience will help to serve as a guide to those wishing to implement this novel technology in their practice, future prospective studies are required to determine optimal dose regimens and treatment schedules.
